# An Empirical Study Analyzing Job Productivity in Toxic Workplace Environments

**DOI:** 10.3390/ijerph15051035

**Published:** 2018-05-21

**Authors:** Amna Anjum, Xu Ming, Ahmed Faisal Siddiqi, Samma Faiz Rasool

**Affiliations:** 1 Glorious Sun School of Business and Management, Donghua University Shanghai, Shanghai 200051, China; naeem@dhu.edu.cn; 2 Business School, University of Central Punjab, Lahore 54600, Pakistan; afsucp50@yahoo.com; 3 School of Management, Shanghai University, Shanghai 200444, China

**Keywords:** toxic workplace environment, workplace ostracism, workplace incivility, workplace harassment, workplace bullying, job burnout, job productivity

## Abstract

*Purpose:* This empirical study aims to determine the effects of a toxic workplace environment, which can negatively impact the job productivity of an employee. *Methodology:* Three hundred questionnaires were randomly distributed among the staff members of seven private universities in Pakistan with a final response rate of 89%. For analysis purposes, AMOS 22 was used to study the direct and indirect effects of the toxic workplace environment on job productivity. Confirmatory Factor Analysis (CFA) was conducted to ensure the convergent and discriminant validity of the factors, while the Hayes mediation approach was used to verify the mediating role of job burnout between the four dimensions of toxic workplace environment and job productivity. A toxic workplace with multiple dimensions, such as workplace ostracism, workplace incivility, workplace harassment, and workplace bullying, was used in this study. *Findings:* By using the multiple statistical tools and techniques, it has been proven that ostracism, incivility, harassment, and bullying have direct negative significant effects on job productivity, while job burnout was shown to be a statistical significant mediator between the dimensions of a toxic workplace environment and job productivity. Finally, we concluded that organizations need to eradicate the factors of toxic workplace environments to ensure their prosperity and success. *Practical Implications:* This study encourages managers, leaders, and top management to adopt appropriate policies for enhancing employees’ productivity. *Limitations:* This study was conducted by using a cross-sectional research design. Future research aims to expand the study by using a longitudinal research design.

## 1. Introduction

In the last few decades, organizations have had a single focus on “profit”, which was based solely on the stock prices. However, the outliers on the other side of the spectrum have been ignored, which are namely the “employees”, despite their established value as the most important assets of an organization [1]. An extensive review of the literature has determined that employees can be categorized as “stars”, who substantially increase organizational output and “toxic workers”, who simply are unsuitable for the organization [2]. Studies have shown that 80% of the issues and concerns regarding employees’ productivity are related to the type of work environment in which they operationalize their assigned tasks [3].

During different time periods, researchers tried to conceptualize the phrase “working environment”. In a simple form, the working environment is the totality of the systems, conditions and situations in which an employee performs his/her tasks [4]. A working environment can be classified into two major spectrums: collaborative workplace environment and toxic workplace environment [5,6,7]. On the positive end of the spectrum, collaborative workplace environments refer to the high-spirit workplace with a community-centered approach in which the employee and employer have an empathetic relationship that fosters the physical and psychological well-being of an employee. On the negative end of the spectrum, toxic workplace environments induce repulsive experiences, which lead to the negative, adverse and reduced outcomes of the employees [8]. A toxic environment is similar to a cancer that damages all the stakeholders of an organization as it creates toxic culture, toxic leaders, and toxic employees, which ultimately create a toxic organization [9]. Toxic behaviors in the workplace can increase the organizational cost due to the loss of a positive company image, low self-esteem, loss of employee morale, high turnover, work life conflict, high absenteeism, poor employee health, and lowered employee productivity [10]. Research has shown that a “toxic workplace environment” damages the organizational outcome. There is need for researchers to explore the root causes and potential consequences of the toxic workplace for both the employees and the whole organization [11,12]. Therefore, this study intends to highlight the different forms of toxic workplace environments and its consequences in the form of high job burnout and low productivity level. Job burnout is treated as a mediating variable between a toxic workplace environment and job productivity. The term ‘toxic workplace environment’ has multiple facets that include: workplace ostracism, workplace narcissism, workplace bullying, workplace incivility, aggressiveness, workplace harassment, workplace passivity, and others [13]. Ferris and Salzburg introduced the four dimensions that defines a toxic workplace, such as ostracism, bullying, incivility, and harassment [14,15]. To eradicate the toxic workplace environment, this study has the following two objectives:

- Determine the direct impact of the dimensions of toxic workplace environment on job productivity.
- Test the mediating role of job burnout between the multifaceted toxic workplace environment and job productivity.

Based on these objectives, the following hypotheses will be tested during this study:

**Hypothesis** **1:** *There is a negative impact of workplace ostracism on job productivity*.

**Hypothesis** **2:** *There is a negative impact of workplace incivility on job productivity*.

**Hypothesis** **3:** *There is a negative impact of workplace harassment on job productivity*.

**Hypothesis** **4:** *There is a negative impact of workplace bullying on job productivity*.

**Hypothesis** **5:** *Job burnout mediates the relationship between workplace ostracism and job productivity*.

**Hypothesis** **6:** *Job burnout mediates the relationship between workplace incivility and job productivity*.

**Hypothesis** **7:** *Job burnout mediates the relationship between workplace harassment and job productivity*.

**Hypothesis** **8:** *Job burnout mediates the relationship between workplace bullying and job productivity*.

## 2. Literature Review

### 2.1. Toxic Workplace Environment

The workplace environment is the totality of the interrelationships of individuals at the workplace, which can be technical, human, and organizational [16,17,18]. The workplace environment can be classified into two major categories: collaborative workplace environment and toxic workplace environment. The collaborative workplace environment yields a sense of happiness, joy, harmony, kindness, politeness, cooperation, and facilitation at the workplace [19,20]. The workplace is toxic when individuals in power are greedy and narcissistic and/or use unfair means to bully, harass, threat, and humiliate others. The toxic workplace can cause anxiety, stress, depression, health problems, absenteeism, job burnout, counterproductive work behavior, and ultimately degrade productivity [8,19].

### 2.2. Workplace Ostracism

Workplace ostracism is the perception of an individual regarding his/her social exclusion by his/her peers and supervisors at the workplace [15,21,22,23,24,25,26]. It creates a work environment with negative consequences for the employee in the form of high job dissatisfaction and high turnover intention [9]. Workplace ostracism includes the deliberate reduction in social networks that impact the physical and psychological health of an individual [14,22,25,26]. Ostracism is a painful experience that can result in negative and stressful outcomes [27,28]. Hobfoll recommended psychological capital as a combating agent to reduce the negative impact of workplace ostracism on an employee’s counter-productive work behavior [29]. In an organizational context, ostracism can create negative work behaviors, such as high anxiety, depression, emotional exhaustion, and lack of job productivity [30]. Overall, workplace ostracism is a stressor that stops an individual from performing their routine workplace activities, reduces their work motivation and adversely affects the productivity of both the individual and organization.

### 2.3. Workplace Incivility

Workplace incivility can be defined as the deliberate intention of one employee to violate the workplace norms by disrupting other employees for his/her personal gain [31,32,33]. Workplace incivility can be the verbal abuse or non-verbal actions of an individual that show disrespect towards colleagues or peers [34]. Due to the infancy of the term ‘incivility’ in the field of management as well as in the education and health sectors, researchers have focused on locating and eradicating its root causes, which negatively impact the employees’ self-esteem, respect, satisfaction, and productivity. Incivility causes physical, psychological and social harm to an individual through isolation, anxiety, depression, mental disability, and the development of low confidence [30]. It is a type of deviant and discourteous behavior with a low intensity that triggers an individual to undermine the image and performance of peers at the workplace. It includes expressing disgraceful, disrespectful, rude and harsh comments to an individual, who may be a peer, colleague or subordinate [35,36,37,38,39].

### 2.4. Workplace Bullying

Workplace bullying negatively impacts employees’ mental health [40]. Bullying includes criticism, blaming, social isolation, humiliation, joking, and excessive monitoring of an employee [36,41,42]. Bullying is a situational and contextual factor that is not only limited to bosses as it can also be exerted by supervisors, managers, peers, subordinates, colleagues and anyone in the workplace [43,44,45,46]. It can be entrenched into organizational settings and culture, which creates detrimental physical and mental health problems, emotional exhaustion and job burnout [47]. The term of ‘bullying’ can refer to individual or organizational bullying. Individual bullying comprises of dispute-related bullying, escalated bullying, complex bullying, delegated bullying, bystander bullying, merry-go-round bullying, gang bullying, good guy/bad guy bullying, subordinate bullying, and personality disorder bullying. Organizational bullying includes external pressure bullying, history and culture bullying, senior team tactic bullying, and process bullying [43,44,48]. Initially, this concept was introduced by Leymann Heinz in 1996 [49] and has been adopted in different organizations, industries, sectors, and countries [50]. As an indicator of a toxic workplace environment, bullying may result in job stressors, job burnout, buffering resources, negative work environment, work destruction, and low productivity [51,52].

### 2.5. Workplace Harassment

Harassment is unwanted conduct, which humiliates an individual; violates an individual’s dignity; or intimidates others [53,54]. Harassment may include unsolicited and explicit speech about race, sex, religion, belief, origin, age, genes, color or ethnicity as a part of a toxic workplace environment [55,56]. This concept was introduced by Farley in 1978 [57] and has gained a considerable amount of attention from researchers since the 1980′s as it is a significant source of stress at the workplace. In some Asian countries, there are limited efforts to investigate any potential harassment as it is considered to be disrespectful, disgraceful, and insulting for the victim [53,58]. Only a few individuals in Pakistan are willing to talk about workplace harassment [59]. Workplace harassment against women has been a frequent subject of studies, while workplace harassment against men rarely receives attention and is not frequently the subject of studies [60]. Males and females of all ages experience workplace harassment. Feminist scholars have linked this concept with gender discrimination, job threats, paradoxical power threat, stereotype thoughts, male dominant society, and illiteracy [61]. Workplace harassment not only negatively impacts the individual but also has the potential to affect the emotional well-being of an entire workplace. This leads to the loss of employee morale, which consequently reduces organizational productivity. Therefore, it is in the company’s best interest to ensure a safe workplace.

### 2.6. Job Burnout

Job burnout is one outcome of a toxic workplace environment that is defined by various dimensions: ostracism, narcissism, cynicism, aggressiveness, bullying, harassment, abusive supervisor, interpersonal conflict, and mistreatment [62,63,64]. This concept was first proposed by Freudenberger in 1975, which was characterized by emotional exhaustion, low motivation, and commitment that ultimately leads towards low productivity [41,65]. Job burnout refers to the emotional detachment of an employee from his/her task, which creates dissatisfaction with personal and professional life, achievements and work-life conflicts [66,67]. A burned-out employee manifests withdrawal behavior through absenteeism, increased leave and constantly being late. This will ultimately affect turnover. Employees who experience job burnout, usually suffer from mental and health problems, including depression, anxiety, tension, stress, work overload, sleeping problems, and muscle pain. This substantially reduces their ability to function in life [45,68,69,70]. Job burnout is basically a syndrome that can be created due to situational and individual factors. This syndrome causes depersonalization, poor self-assessment, self-underestimation, high stress, and negative job outcomes [71,72,73,74].

### 2.7. Job Productivity

The term of ‘productivity’ involves measuring the efforts of an individual to effectively and efficiently convert the input resources into output [75]. It basically refers to the time spent on the desired activity, which the employee is expected to perform within specific limited resources [76]. Scholars argued that the term of ‘productivity’ has no single operational definition as it can vary according to the context, culture, and type of the organization [41]. Job productivity integrates both the concepts of employee productivity and organizational productivity, which can be measured by quality considerations [77]. Job productivity depends upon multiple factors, including: individual ability, working environment, HR motivational policies, support from supervisors, and organizational standards. It can be measured in monetary terms, which has the attributes of financial, human, organizational, and social capital [59,78]. The level of productivity does not only depend upon the individual’s ability but also on his/her social network and work environment. Employees who enjoy their work environments are more engaged, more productive, happier, and healthier. Therefore, it makes perfect sense to generate a workplace that is conducive to the well-being of the workforce and organizations should make efforts to provide a better environment for employees so that they may feel comfortable and committed to their jobs in order to increase productivity.

## 3. Data and Methodology

### 3.1. Sample and Procedure

A survey design was used to collect quantitative data, before rigorous data analytical techniques were applied to test the nature of the relationships among the selected variables. This study aimed to determine the impact of different dimensions of a toxic workplace environment, including ostracism, incivility, harassment, and bullying, on job productivity. Furthermore, we wanted to test the mediation of job burnout between toxic workplace environments and job productivity. A positivism paradigm was favored to test these relationships by using a survey questionnaire design recommended by Robson & McCartan [79]. A systematic sampling technique was used to select seven private universities in the city of Lahore, Pakistan [80]. Employees were the unit of analysis for this study. A total of 300 questionnaires were randomly distributed among staff members and 267 responses were received back. Thus, the response rate was 89%. For analysis purposes, AMOS 22 was used to conduct CFA and to verify the direct and indirect effect of variables. SPSS was used for descriptive statistics, while the Hayes mediation [81] approach was used to verify the mediating role of job burnout between toxic workplace environments and job productivity.

### 3.2. Measurements

A toxic workplace environment consists of four sub-constructs: ostracism, incivility, harassment, and bullying. All these constructs were measured on a five-point Likert scale adapted from the ostracism scale [82], incivility scale [83], harassment scale [84], bullying scale [85], job burnout scale [86], and job productivity scale [87].

## 4. Empirical Findings/Analysis

### 4.1. Confirmatory Factor Analysis (Measurement Model)

Confirmatory factor analysis (CFA) was conducted to judge the convergent and discriminant validity of each construct and to determine the fitness of the overall measurement model. AMOS 22 was used to conduct CFA. Table 1 revealed the results of convergent validity, which showed that all factor loadings were greater than 0.60 and the composite construct reliability was also greater than the threshold value of 0.70. The average variance extracted (AVE) of all constructs was also greater than the minimum recommended value of 0.50, which indicated that our six-factor CFA, met the standards of convergent validity. Table 2 presented the overall fitness of the six factor CFA which indicated a moderate fit of the indices with the data for direct effects (GFI = 0.937, AGFI = 0.861, NFI = 0.930, TLI = 0.910, CFI = 0.966, & RMSEA = 0.031) and for indirect effects or the mediation model. The discriminate validity was estimated by using the typologies mentioned in literature by Fornell and Hair et al. [88,89]. Table 3 shows the results of the discriminant validity in which the AVE of all conducts were greater than maximum shared square variance (MSV) and average shared square variance (ASV). The square root of AVE of each construct was also greater than its correlation, thus supporting our measurement model of discriminant validity.

### 4.2. Descriptive Statistics

From our total sample (267 cases), 211 (79%) were males, and 56 (21%) were females. In terms of age, 158 (59%) were under 30 years, 52 (19.4%) were 30–39 years old, 36 (13.5%) were 40–49 years old, and 21 (8.1%) were 50–59 years old. This showed that most of the respondents were under the age of 30 years. In terms of qualification, 28 (10.8%) respondents had a PhD degree, 102 (38.9%) respondents had a Master of Philosophy degree, 62 (23.5%) had a Master degree, 70 (26.5%) had a Bachelor degree and 5 (1.9%) had intermediate degrees. Among 267 respondents, 243 (89%) were full-time employees and only 24 (11%) were contractual employees. For salary, 61.2% of the respondents had a salary less than 30,000 PKR (Pakistani Rupee), 18% had a salary range of 30,000–50,000 PKR, 11.1% had a salary range of 51,000–80,000 PKR, and only 9.7% had a salary over 80,000 PKR.

Table 4 shows the results of the minimum, maximum, means, and standard deviations of the data. In this research study, a survey of 38 items was used and the response rate of all items vary from 1 to 5. The results showed that the mean values of different items were 3.34–3.64 and the standard deviation was 0.889–1.064.

### 4.3. Regression Analysis

The direct and indirect effects were tested by using the structural equation modeling technique (SEM). Table 5 displayed the results of the direct effects of four constructs of toxic workplace environments (ostracism, incivility, harassment, and bullying) on job productivity.

The regression coefficient of ostracism was -0.884. Since the regression coefficient was negative with significant at the 0.05 level, this supported our hypothesis H1, which stated that there is a negative significant impact of work-place ostracism on job productivity. Our results showed that greater ostracism resulted in less job productivity.

Similarly, the regression coefficient of incivility was −0.274 and was significant at the 0.05 level. This also supported our hypothesis H2, which stated that there is a negative significant impact of workplace incivility on job productivity.

Furthermore, the regression coefficient of harassment was also negative (−0.783), which was significant at the 0.05 level. Therefore, we can conclude that there is a negative significant impact of workplace harassment on job productivity, which supports our hypothesis H3.

Likewise, the regression coefficient of bullying was −0.696, which was also significant at the 0.05 level. Therefore, it supported our hypothesis H4, which stated that there is a negative significant impact of workplace bullying on job productivity. Thus, job productivity will decrease if workplace bullying exists.

We employed the Bootstrapping technique to test the indirect effects among variables. Table 6 shows the results of the indirect effects.

As the indirect effect of ostracism on job productivity through job burnout was 0.229, which was significant at 0.05 level, this supported our hypothesis H5, which stated that job burnout mediates the relationship between workplace ostracism and job productivity.

Similarly, the indirect effect of incivility on job productivity through job burnout was 0.271, which was significant at the 0.05 level. This supported our hypothesis H6, which stated that job burnout mediates the relationship between workplace incivility and job productivity.

In the same way, the indirect effect of harassment on job productivity through job burnout was 0.314, which was significant at the 0.05 level. This supported our hypothesis H7, which stated that job burnout mediates the relationship between workplace harassment and job productivity.

Finally, the indirect effect of bullying on job productivity through job burnout was 0.329, which was significant at the 0.05 level. This supported our hypothesis H8, which stated that burnout mediates the relationship between workplace bullying and job productivity.

**Figure 1:**
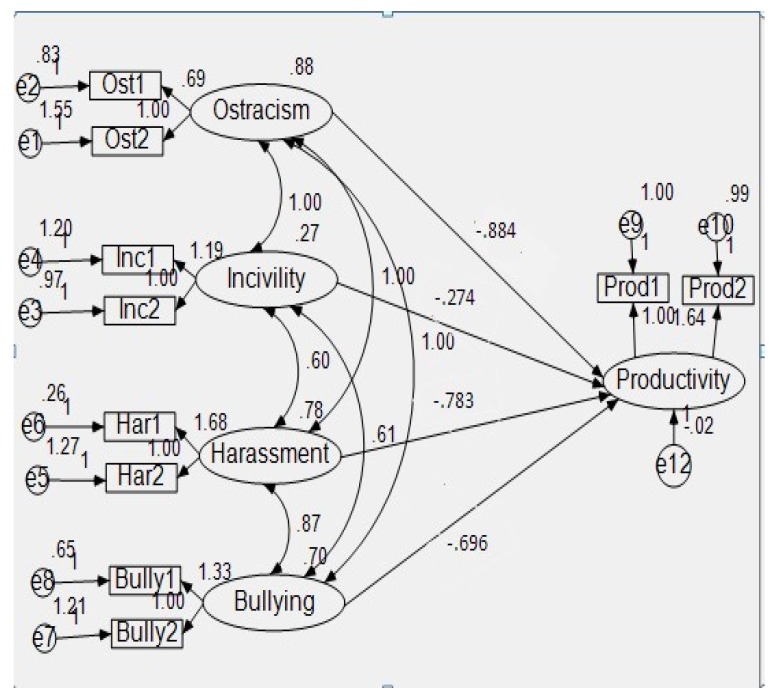
Path Analysis-I.

**Figure 2:**
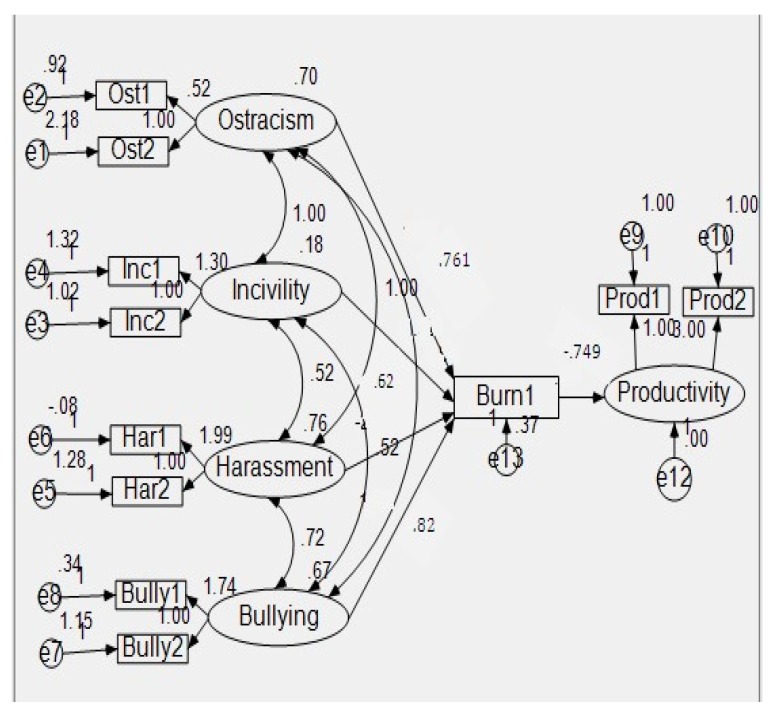
Path Analysis-II.

Path Analysis-I (Figure 1) was conducted to evaluate the overall goodness of fit. A Chi-square value that is close to zero indicates little difference between the expected and observed covariance matrices with a probability level greater than 0.05, justifying the absence of meaningful unexplained variance. Moreover, to estimate a better goodness of fit, due to the fact that Chi-square is sensitive to sample size, we calculated the ratio of Chi-square to degrees of freedom, which should be less than three in an acceptable data-model fit. In addition, we utilized the Comparative Fit Index (CFI) [90], the Tucker–Lewis Index (TLI) [91], the Root Mean Square Error of Approximation (RMSEA) [92], and the Standardized Root Mean Square Residual (SRMR) [93]. The indicators of a well-fitting model are evidenced by CFI and TLI that are greater than 0.95, RMSEA that is less than 0.06, and SRMR that is less than 0.08 [9]. The mediated regression in the Path Analysis-II also confirms all the above mentioned calculations, thus providing good evidence about the good fit of the model (Figure 2).

### 4.4. Mediation Analysis

The Hayes mediation [81] approach was used to check the mediating role of job burnout between the four dimensions of a toxic workplace environment and job productivity. The dimensions of toxic workplace environments were ostracism, incivility, harassment, and bullying, while job productivity was a dependent variable. For mediation, we had to ensure three conditions: whether mediation existed or not (checked by *p*-value); the effect of mediation (by value of effect); and whether mediation was statistically significant or not. From the output in Table 7, it could be observed that job burnout acted as a mediator between ostracism and job productivity (H5: effect = 0.240); incivility and job productivity (H6: effect = 0.601); harassment and job productivity (H7: effect = 0.415); and also served as a mediator between bullying and job productivity (H8: effect = 0.112). As the values of the effects are greater than zero for all four combinations of variables and the p-values were also less than 0.05 (standard sig. value), we concluded that the mediation of job burnout existed between the variables. The values of both BootLLCI and BootULCI had negative signs, which proved that job burnout acted as a statistically significant mediator between all four dimensions of toxic workplace environment and job productivity.

## 5. Discussion

A toxic workplace environment can create difficulty in an employee’s work life and can reduce his/her job performance. This present study aimed to determine the direct influence of ostracism, incivility, harassment, and bullying (dimensions of toxic workplace environment) on job productivity and also tried to analyze the indirect effects of these variables with job burnout being a mediator. By using the multiple statistical tools and techniques, it has been proven that ostracism, incivility, harassment, and bullying have direct significant negative effects on job productivity, which was shown by the negative coefficients of −0.884, −0.274, −0.783, −0.696 respectively, (*p* < 0.05). For indirect effects, job burnout was shown to be a statistically significant mediator between the four dimensions of toxic workplace environment and job productivity, which was confirmed by the validation of the hypotheses. Our results clearly justify that a toxic workplace has direct significant negative effect on the job productivity of an employee. Consistent with prior research, this study also shows that workplace ostracism [94], workplace incivility [34], workplace harassment [53,58], and workplace bullying [43,44,46] reduce job productivity. Previous studies also indicated that a toxic workplace increased job burnout [24,95].

Unfortunately, toxic workplaces exist in many organizations and are generally characterized by a culture of dysfunctional interpersonal dynamics despite of the awareness that human capital is the contributing factor for any organization’s sustainable growth and innovation. Mostly, organizational or corporate culture is driven from the top-down approach, and if the leadership is not concerned about the toxic environment, it can be difficult to shift the culture. There are several approaches to address this issue. One of them is for the organization leaders to demonstrate their support to employees by acknowledging their difficulties at work and providing necessary support, especially for the tasks that have more demanding requirements. When the employees have a sense of social support, appreciation, and a positive work environment, they perform better.

We have included a few suggestions for minimizing if not eradicating the toxic culture in a work environment:

1. One can do a self-assessment: “Are my actions or performances contributing towards a positive environment?”
2. Actively disengage from negative interactions.
3. Try to focus on turning a bad situation into a good learning experience. Frequently, the strongest personal growth comes from thriving on the most difficult situations. When an employee is working in a toxic environment, he/she should try to pay close attention to the lessons he/she can take away from the experiences. In every adversary, there is positive insight that one can learn to become a better person.
4. Communicate positive messages to others. Employees should share appreciation for peers, team members, subordinates, and also for the work they do. Recognize that people like to feel appreciated in different ways.
5. Establish and implement clear policies and communication procedures that address toxic factors, such as harassment and bullying. Most companies have a code of conduct policies, but many of those policies are general or solely address unethical and financial misconduct. Companies rarely maintain policies with specific language that adequately defines a range of prohibited behaviors. A sound policy should be established with clear and multiple reporting mechanisms in place.
6. Once the policy has been established, the leaders must ensure all managers and employees receive the training on how to identify, respond, and report these toxic behaviors. Training must also highlight the challenges and fears of employees who struggle to report these types of behaviors.
7. Even though one may work in a really toxic environment, an individual should try to not add to the toxicity of the work environment and instead should try to be of benefit in removing the toxic factors from the workplace.

## 6. Conclusions

Productivity enhancement is a major apprehension of every organization across the globe irrespective of the organization’s nature, operations, functions, area, and sector. However, the meaning and sense of the term ‘productivity’ vary according to the vision and objectives of the organization. Furthermore, there are factors that play an important role in cultivating the productivity in different organizations according to their internal cultures and environments. From an extensive review of the academic literature available on the topic, it has been identified that organizational productivity is conditional on the level of their employee’s productivity. The researchers attempted to explore factors that could affect the intensity of productivity. As evident from the results, a toxic work environment significantly impacts the job productivity and the job burnout. Thus, we concluded that the toxic workplace increases the job burnout level of an employee. When an employee feels negatively about the organization, he/she tends to compromise the productivity level of his/her performance, which could also increase the stress level of an employee. This study recommends that in every organization, HR departments and policy makers should develop and implement strict policies for eradicating a toxic workplace environment to make it collaborative and conducive for the employees.

## Figures and Tables

**Table 1:** Results of confirmatory factor analysis and Convergent validity and construct reliability.

Variables	Measurement Items	Standard Loadings	AVE	CCR	Cronbach Alpha
Ostracism	Ost1 Ost2	0.823 0.914	0.786	0.802	0.908
Incivility	Inc1 Inc2	0.692 0.721	0.531	0.715	0.912
Harassment	Har1 Har2	0.827 0.912	0.623	0.818	0.904
Bullying	Bully1 Bully2	0.835 0.916	0.623	0.721	0.834
Job burnout	Burnout	0.935	0.521	0.857	0.949
Job productivity	Prod1 Prod2	0.898 0.689	0.518	0.597	0.872

**Table 2:** Model Fitness.

	Direct Effect	Indirect Affect
GFI	0.937	0.941
AGFI	0.861	0.893
NFI	00.930	0.925
TLI	0.910	0.931
CFI	0.966	0.955
RMSEA	0.031	0.049

**Table 3:** Discriminant Reliability.

	AVE	MSV	ASV	Ost_all	Inc_all	Har_all	Bully_all	Burnout_all	Prod_all
Ostracism_all	0.528	0.524	0.222	0.773					
Incivility_all	0.701	0.214	0.186	0.376	0.849				
Harassment_all	0.521	0.381	0.392	0.554	0.542	0.707			
Bullying_all	0.623	0.331	0.218	0.307	0.497	0.609	0.808		
Job burnout_all	0.664	0.318	0.252	0.460	0.361	0.572	0.536	0.784	
Job productivity_all	0.526	0.514	0.265	0.710	0.257	0.609	0.199	0.453	0.766

Note: Diagonal value: Square root of AVE and Non-diagonal value: correlation.

**Table 4:** Results of Descriptive Statistics.

Name of Variables	N	Min.	Max.	Mean	Std. D.
Ostracism	267	1	5	3.42	1.026
Incivility	267	1	5	3.40	1.064
Harassment	267	1	5	3.34	0.944
Bullying	267	1	5	3.40	0.889
Job burnout	267	1	5	3.64	1.024
Job productivity	267	1	5	3.47	0.999

**Table 5:** Results of Direct Effects.

Hypothesis Tested	Independent Variables	Dependent Variables (Job Productivity)		Remarks
		*β* Coefficients	*p*-Value	
H1	Ostracism	−0.884	0.000	Significant
H2	Incivility	−0.274	0.010	Significant
H3	Harassment	−0.783	0.002	Significant
H4	Bullying	−0.696	0.031	Significant

Note: All values were significant at 0.05 significance level (two-tailed).

**Table 6:** Results of Indirect Effects.

Hypothesis Tested	Independent Variables	Dependent Variable (Job Productivity)		Remarks
		β Coefficients	*p*-Value	
H5	Ost → Burnout → Prod	0.229	0.0	Significant
H6	Inc → Burnout → Prod	0.271	0.0	Significant
H7	Har → Burnout → Prod	0.314	0.0	Significant
H8	Bully → Burnout → Prod	0.329	0.0	Significant

**Table 7:** Results of Indirect Effect (Mediation Effect).

Hypothesis Tested	Paths	Effect	*p*-Value	Boot LLCI	Boot ULCI	Remarks
H5	Ost → Burnout → Prod	0.240	0.000	−0.1093	−0.0084	Statistically significant mediation
H6	Inc → Burnout → Prod	0.601	0.001	−0.0752	−0.0078	Statistically significant mediation
H7	Har → Burnout → Prod	0.415	0.031	−0.0548	−0.0281	Statistically significant mediation
H8	Bully → Burnout → Prod	0.112	0.000	−0.0266	−0.1435	Statistically significant mediation
